# Impact of Coronavirus Disease 2019 on Clinical Characteristics in Patients With Lung Cancer: A Large Single-Centre Retrospective Study

**DOI:** 10.3389/fonc.2021.693002

**Published:** 2021-08-16

**Authors:** Yu Zhang, Jie Li, Zhi-Ke Li, Xiyue Yang, Jie Bai, Lihua Liu, Tangzhi Dai, Gang Feng, Qiu Li, Xiaobo Du

**Affiliations:** ^1^Department of Medical Oncology, Cancer Center, West China Hospital of Sichuan University, Chengdu, China; ^2^Department of Oncology, MianYang Central Hospital, MianYang, China; ^3^Department of Oncology, Affiliated Hospital of North Sichuan Medical College, Nan Chong, China; ^4^Department of Rehabilitation Medicine, MianYang Central Hospital, MianYang, China

**Keywords:** COVID-19, lung cancer, influence, CT screening, clinical characteristics

## Abstract

Lung cancer is the most common cancer malignancy worldwide. With the continuous spread of the coronavirus disease 2019 (COVID-19) globally, it is of great significance to explore the impact of this disease on the clinical characteristics of lung cancer. Thus, we aimed to investigate whether the COVID-19 pandemic had any influence on the clinical characteristics and diagnosis of patients with lung cancer. We collected clinical and demographic data of patients who were newly diagnosed with lung cancer at our hospital between February 2019 and July 2020. Overall, 387 patients with lung cancer were divided into two groups for analysis: epidemic group (from February to July 2020) and pre-epidemic group (from February to July 2019). The source of diagnosis and clinical characteristics of the two groups were analysed. T-test and Mann-Whitney U were used for continuous variables, and Chi-squared or Fisher’s exact test for categorical variable. We found that during the epidemic period, 110 cases of lung cancer were incidentally diagnosed during COVID-19 screening, accounting for 47.6% of all newly diagnosed lung cancer cases at our hospital. The proportions of patients who were diagnosed based on symptoms and physical examination in the epidemic group were 34.2 and 18.2%, respectively, while that in the pre-epidemic group were 41.7 and 58.3%, respectively. There was significant difference in the source of diagnosis between the two groups. In a subgroup analysis of the epidemic group, the average tumour volume of the patients diagnosed with COVID-19 screening was significantly smaller than that of the patients diagnosed with symptoms and physical examination. In conclusion, the continuation of the COVID-19 pandemic may impact the screening and clinical characteristics of lung cancer and require large-scale and longer-term observation.

## Introduction

Lung cancer is a serious threat to human health, having the highest morbidity and mortality rates among cancers worldwide (1). The continuous spread of the coronavirus disease 2019 (COVID-19) has made the global public health system encounter unprecedented challenges and created great psychological pressure for the general public (2–4). Therefore, adequate management of patients with lung cancer during the COVID-19 pandemic is particularly important in the context of its long-term persistence (5, 6). As high-risk areas, hospitals in China initiated special control measures to prevent nosocomial infections during the COVID-19 pandemic. Although the prevention and control measures employed in the hospitals have had positive effects in controlling the spread of the virus, they have also affected the treatments for other conditions, including lung cancer. However, the association between COVID-19 and the diagnosis and clinical characteristics of lung cancer is unclear. Thus, this study aimed to identify and analyse any influences of COVID-19 on the clinical characteristics and diagnosis of newly confirmed lung cancer patients during the pandemic in our hospital.

## Materials and Methods

**Figure 1:**
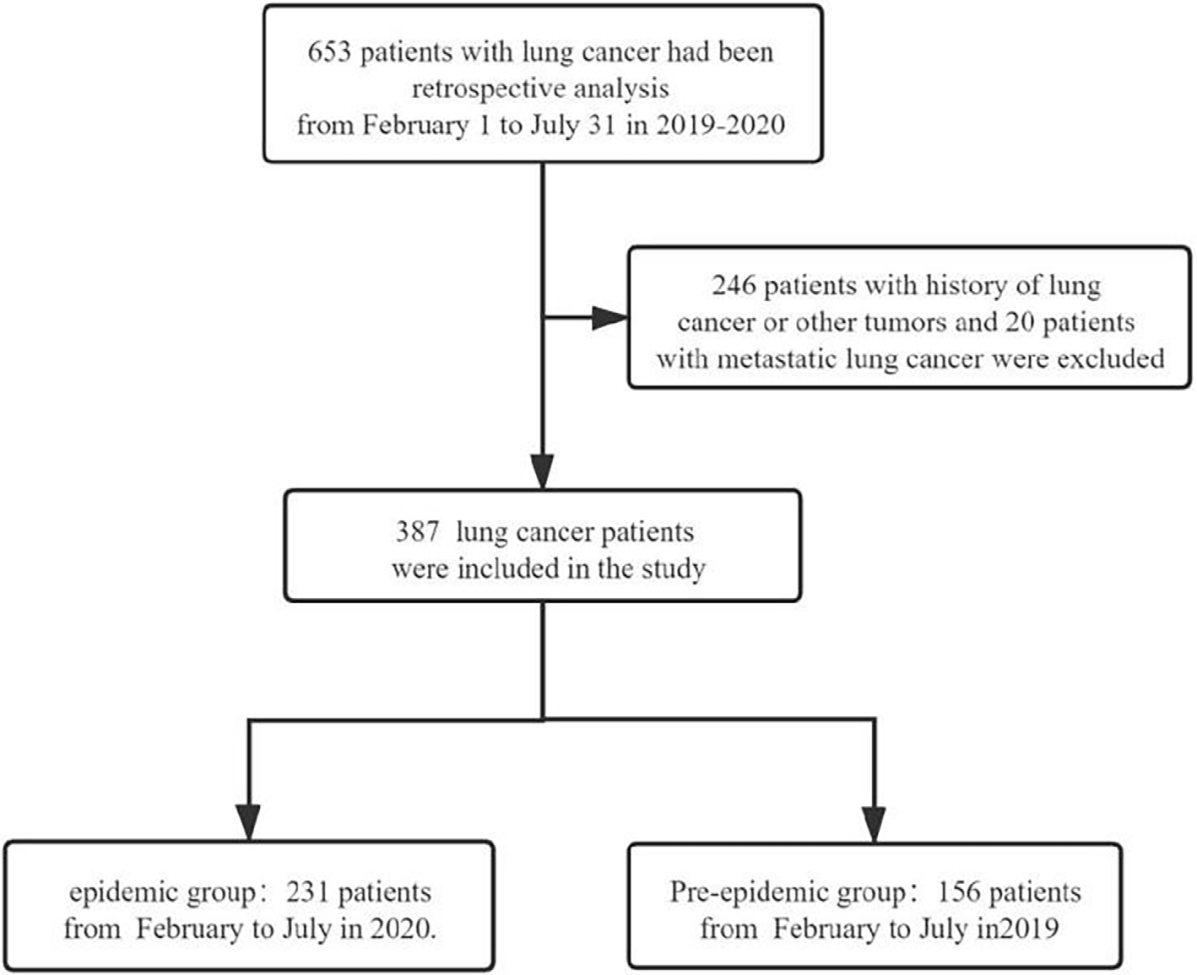
Flow chart for patient inclusion in the study.

In this single-centre retrospective study, we reviewed the data of patients with lung cancer in Mianyang Central Hospital between February 2019 and July 2020. This hospital is a large regional medical centre with 3,000 inpatient beds. In 2019, there were more than 2.4 million outpatient visits and 100,000 inpatient visits, covering a population of nearly 20 million. The inclusion criteria were as follows (1): age ≥18 years (2); pathologically confirmed diagnosis, including non-small-cell lung cancer (squamous cell carcinoma and adenocarcinoma) and small-cell lung cancer (3); evaluable lesions in the lungs; and (4) hospitalisation. Exclusion criteria were as follows: (1) previous history of other tumours, pulmonary metastatic disease, or lung cancer; (2) presence of lymphoma, thymoma, or other mediastinal tumours; and (3) patients without pathological diagnosis. **Figure 1** depicts the process of patient selection. This study was approved by the Medical Ethics Committee of Mianyang Central Hospital (No: S-2021-025). The patients who were newly diagnosed with lung cancer were divided into the epidemic and pre-epidemic groups based on their time of diagnosis in relation to the COVID-19 epidemic in China. The Chinese government started a first-level response to public health emergencies on January 23, 2020, after the outbreak of COVID-19. Therefore, we defined patients in the epidemic group as those who were newly diagnosed with lung cancer between February and July 2020, and patients in the pre-epidemic group as those who were newly diagnosed with lung cancer between February and July 2019. The endpoint was confirmation of lung cancer through surgery or biopsy.

The source of diagnosis (reason for hospitalisation) and clinical characteristics, including sex, age, stage, Vt (tumour volume), tumour site, pathological category, interval time [time interval between computed tomography (CT) finding of lung mass and pathological diagnosis], and duration of hospital stay of the two groups, were analysed in this study. The sources of diagnosis were divided into three categories: physical examination, symptoms (cough, expectoration, haemoptysis, dyspnoea, chest pain), and COVID-19 screening. The number of outpatients and those who underwent general physical examinations were expected to affect the further quantity of newly diagnosed lung cancer patients. Therefore, we compared such numbers in the two corresponding periods. Staging was carried out according to the American Joint Committee on Cancer/International Union Against Cancer 2018 version of lung cancer staging standards. SPSS 24.0 software (IBM SPSS Statistics for Windows, Version 24.0. Armonk, NY, USA: IBM Corp.) was used for statistical analyses. Chi-squared or Fisher’s exact test for categorical variable, and the independent-sample t-test (if normal distribution) or Mann-Whitney U (if non-normal distribution) were used to analyse continuous variable, to compare the epidemic and pre-epidemic groups.

## Results

A total of 387 patients with newly diagnosed lung cancer were included in this study. There were 231 and 156 patients in the epidemic and pre-epidemic groups, respectively. As shown in **Table 1**, there was no significant statistical difference in the clinical stage of the two groups (*p* = 0.364). There were also no significant statistical differences in sex, tumour site, pathology, interval time, tumour volume, and duration of hospital stay. However, there were statistically significant differences between the two groups in age (*p* = 0.018) and the source of diagnosis (*p* < 0.001). In the epidemic group, the source of diagnosis for 110 patients was “COVID-19 screening,” accounting for 47.6% of the total number of patients diagnosed at that time. The sources of diagnosis for the other patients were physical examination (79, 34.2%) and symptoms (42, 18.2%). However, in the pre-epidemic group, 91 (58.3%) and 65 (41.7%) patients were diagnosed from physical examination and symptoms, respectively. From February to July 2020, the monthly average number of outpatients and physical examination patients also decreased significantly compared with the same period in 2019.

**Table 1:** The clinical characteristics of newly diagnosed lung cancer patients between the epidemic groups and the pre-epidemic groups.

Clinical characteristics		Epidemic group (January to July in 2020) (n = 231)	Pre-epidemic group (January to July in 2019) (n = 156)	P value
Sex	Male	108 (46.8%)	70 (44.9%)	0.716
	Female	123 (53.2%)	86 (55.1%)	
Age (y)	mean ± SD	56.3 ± 10.8	59.0 ± 10.9	0.018
Pathology	SCC	26 (11.3%)	21 (13.5%)	0.388
	AC	195 (84.4%)	132 (84.6%)	
	SCLC	10 (4.3%)	3 (1.9%)	
Tumour site	Left	99 (42.9%)	70 (44.9%)	0.814
	Right	125 (54.1%)	80 (51.3%)	
	Multiple lesions	7 (3.0%)	6 (3.8%)	
Source of diagnosis	COVID-19 screening	110 (47.6%)	0 (0.0)	<0.001/0.239^&^
	Physical examination	79 (34.2%)/(65.3%)^*^	91 (58.3%)	
	Symptoms	42 (18.2%)/(44.7%)^*^	65 (41.7%)	
Interval time (m)	Median (IQR)	1.0 (4.5)	1.0 (2.5)	0.051
Stage	IA1	44 (19.0%)	23 (14.7%)	0.729
	IA2	90 (39.0%)	56 (35.9%)	
	IA3	31 (13.4%)	21 (13.5%)	
	IB	17 (7.4%)	18 (11.5%)	
	II	20 (8.7%)	14 (9.0%)	
	III	20 (8.7%)	16 (10.3%)	
	IV	9 (3.8%)	8 (5.1%)	
Vt (cm3)	Median (IQR)	1.0 (4.7)	1.3 (7.3)	0.285
Hospital stay (d)	mean ± SD	11.8 ± 6.6	11.8 ± 7.3	0.993
No. of Outpatients (n/month)	mean ± SD	142,425 ± 36,613	194,549 ± 15,104	0.009
No. of physical examination patients (n/month)	mean ± SD	2,574 ± 1,653	4,548 ± 699.1	0.02

SCC, squamous cell carcinoma; AC, adenocarcinoma; SCLC, small-cell lung cancer; y, year; m, months; d, days; Vt, tumour volume; Interval time, the time interval between CT finding of lung mass and pathological diagnosis; SD, std. deviation; IQR, interquartile range; *The previous number was the percentage of patients from three diagnostic sources including COVID-19 screening (n = 231), the latter number was the percentage of patients who were excluded from the diagnostic source of COVID-19 screening (n = 121). ^&^The former p value is the result of the comparison of the three groups, and the latter p value is the result of the comparison of the two groups after excluding the patients whose diagnosis source is COVID-19 screening.

In the subgroup analysis of the epidemic group, we analysed the clinical characteristics of patients from different diagnostic sources (**Table 2**). The results showed that the average tumour volume of patients with “COVID-19 screening” was significantly smaller than that of the patients with other diagnostic sources (*p* = 0.002). There were no significant statistical differences in sex, age, pathology, interval time, stage, tumour site, and duration of hospital stay.

**Table 2:** The clinical characteristics of epidemic subgroup.

Clinical characteristics		Diagnostic source with COVID-19 screening (n = 110)	Diagnostic source physical examination and symptoms (n = 121)	*P* value
Sex	Male	53 (48.2%)	55 (45.5%)	0.678
	Female	57 (51.8%)	66 (54.5%)	
Age (y)	mean ± SD	55.4 ± 10.4	57.1 ± 11.2	0.249
Pathology	SCC	9 (8.2%)	17 (14.0%)	0.615
	AC	98 (89.1%)	97 (80.2%)	
	SCLC	3 (2.7%)	7 (5.8%)	
Tumour site	Left	42 (38.2%)	57 (47.1%)	0.072
	Right	62 (56.4%)	63 (52.1%)	
	Multiple lesions	6 (5.5%)	1 (0.8%)	
Interval time (m)	Median (IQR)	0.8 (1.5)	2.0 (8.1)	0.436
Stage	IA_1_	14 (12.7%)	30 (24.8%)	0.068
	IA_2_	48 (43.6%)	42 (34.7%)	
	IA_3_	18 (16.4%)	13 (10.7%)	
	IB	11 (10.0%)	6 (5.0%)	
	II	7 (6.4%)	13 (8.7%)	
	III	7 (6.4%)	13 (8.7%)	
	IV	5 (4.5%)	4 (3.4%)	
V_t_ (cm^3^)	Median (IQR)	1.1 (3.6)	2.8 (5.5)	0.002
Hospital stay (d)	mean ± SD	11.8 ± 7.0	11.8 ± 6.4	0.983

SCC, squamous cell carcinoma; AC, adenocarcinoma; SCLC, small-cell lung cancer; y, year; m, months; d, days; V_t_, tumour volume; SD, std. deviation; IQR, interquartile range.

## Discussion

The COVID-19 pandemic has brought with it serious challenges to China’s medical systems. Especially in the early stages of the epidemic, lack of medical resources, public panic, and insufficient awareness of the virus were common phenomena. In this context, general medical activities were seriously affected. The diagnosis and treatment of various acute and chronic diseases, including cancer and cardiovascular and cerebrovascular diseases, have been restricted to a certain extent (7–9). A British report showed a significant increase in the number of deaths from non-COVID-19 causes compared with the same period in the past (10). The influence of COVID-19 on cancer patients has also attracted researchers’ attention (11). A recent report on the impact of COVID-19 on cancer diagnosis showed that the number of newly registered cancer patients in the Dutch National Cancer Registry decreased by about 25% in the period from March to May 2020 compared with the same period in the past (12).

Lung cancer is one of the most common malignancies. However, the effect of COVID-19 on the clinical characteristics of patients with lung cancer is not clear. Our research showed that due to COVID-19, the average monthly number of outpatients and patients who received physical examination in our hospital decreased significantly from February to July 2020 compared with that in the same period in 2019. Firstly, after the health authorities launched a first-level response to public health emergencies, the hospital entered a state of emergency and strict prevention and control measures were deployed to prevent nosocomial infections. This increased the complexity of treatment administration along with an increase in the difficulty of patients to access the hospital. Moreover, a psychological panic was prevalent among the public about the continued spread of COVID-19. Therefore, the public was worried about cross-infections in hospitals, as they were high-risk areas. In particular, patients who were asymptomatic or had mild symptoms delayed their visit to a medical facility. The above two factors led to a significant decrease in the monthly average number of outpatients and patients receiving physical examination during the epidemic. This in turn was not conducive to the diagnosis of lung cancer. A study in Taiwan showed that during the severe acute respiratory syndrome epidemic in 2003, about 64% of the patients with lung cancer who participated in clinical trials were reluctant to visit the hospital from fear of contracting the infection, and about 4% of the patients decided to stop all treatments (13). Similarly, findings from the United Kingdom show a significant decline in the referral of possible cancer patients during the COVID-19 pandemic (14, 15). Our results showed that the total number of patients diagnosed from physical examination and symptoms in the epidemic group decreased from 156 to 121 compared with the pre-epidemic group. Although there was no significant difference in source of diagnoses between the two groups if only considering physical examination and symptoms. These results show that the persistence of the epidemic is unfavourable for the diagnosis and treatment of patients with cancer.

However, the impact of the COVID-19 epidemic is multifaceted, and our study has observed an interesting phenomenon. CT scan is an important method for the screening of both COVID-19 and lung cancer (16–18). Especially in the early stage of the epidemic, from January to April 2020, the detection of severe acute respiratory syndrome coronavirus 2 nucleic acid was not popularised. Therefore, chest CT screening was required for all hospitalised patients, outpatients, patients with fever, close contacts of patients with COVID-19, and some individuals in the general population at high risk of COVID-19 infection. Interestingly, CT screening for COVID-19 unexpectedly found lung masses in some patients, which was confirmed as lung cancer after further examination. A total of 110 patients with lung cancer were unexpectedly diagnosed through COVID-19 screening, accounting for 47.6% of all patients in the epidemic group. Thus, the number of newly diagnosed lung cancer cases increased by 48.1% during the epidemic (231 *vs.* 156), even as the number of outpatients and patients who received physical examination significantly decreased compared with the same period in 2019. Unexpectedly, the epidemic has led to more patients with lung cancer being detected earlier, and provided an opportunity for early diagnosis and treatment of these patients.

When we analysed the clinical characteristics of the epidemic and pre-epidemic groups, we found that the proportion of stage I–II lung cancer in the epidemic group was 87.5% and in the pre-epidemic group was 84.6%. There was no significant difference between the two groups. However, a study in South Korea showed that the proportion of patients with advanced non-small-cell lung cancer increased during the COVID-19 pandemic (19). In general, patients who are diagnosed with lung cancer by symptoms may already have an advanced stage of the disease than those who are diagnosed by physical examination. In this study, the proportions of patients in the epidemic group who were diagnosed with lung cancer by physical examination (34.2%) and those by symptoms (18.2%) were significantly lower than those in the pre-epidemic group (58.3 and 41.7%, respectively). The high proportion (89.1%) of patients with stage I–II disease observed in our study was due to the unexpected diagnoses of lung cancer in 110 patients during COVID-19 screening. Thus, the diagnoses of these patients by COVID-19 screening played a crucial role in staging composition. Therefore, we did not observe any changes in the composition of staging. In addition, only 42 patients were diagnosed by symptoms, accounting for 18.2% of patients in the epidemic group, which had limited impact on the staging structure. Furthermore, the disease progression of patients with early lung cancer was relatively slow, and the observation time was only 6 months. It is possible that the long duration of the epidemic will have a more significant impact on the staging composition of patients. We also observed a significant difference in the patient age between the epidemic and the pre-epidemic groups. The average age of the 110 lung cancer patients diagnosed by COVID-19 screening in the epidemic group was 55.5 ± 10.4. Excluding these 110 patients, the average age of the remaining 121 patients in the epidemic group was 57.1 ± 11.1, and there was no significant difference compared to the pre-epidemic group (*P* = 0.157). This result shows that more young asymptomatic patients are diagnosed with lung cancer after CT screening. This may be the reason why the age of the epidemic group is younger than that of the pre-epidemic group. In the subgroup analysis of the epidemic group, the patients diagnosed by COVID-19 screening had a trend for earlier stage disease and smaller average tumour volume compared with other patients within the group. These differences were likely due to COVID-19 screening, which resulted in 110 patients with lung cancer being detected earlier. As a result, we observed younger age and smaller tumour volume in the epidemic group.

Our study has some limitations. As a retrospective study, the data were not sufficiently comprehensive, and we only included some clinical features. In addition, the endpoint was confirmation of lung cancer through surgery or biopsy; thus, we were only able to discuss the short-term impact of the COVID-19 epidemic on lung cancer. Because of the short follow-up time, the treatment and prognosis of patients were not included in this study for a more comprehensive analysis. The long-term impact of the COVID-19 pandemic still requires further research.

## Conclusion

In conclusion, the continuation of the COVID-19 epidemic impacted the screening of early-stage lung cancer. Conversely, as COVID-19 screening inadvertently discovered some patients with lung cancer, these patients had the opportunity to receive early diagnosis and treatment. There were no effects on the tumour stage. The continuation of the COVID-19 epidemic may have a long-term impact on the clinical characteristics of lung cancer, requiring large-scale and longer-term observation.

## Data Availability Statement

The original contributions presented in the study are included in the article/supplementary material. Further inquiries can be directed to the corresponding authors.

## Ethics Statement

The studies involving human participants were reviewed and approved by the Medical Ethics Committee of Mianyang Central Hospital. Written informed consent for participation was not required for this study in accordance with the national legislation and the institutional requirements.

## Author Contributions

Guarantor of integrity of the entire study: XD and QL. Study concepts and design: XD and QL. Literature research: YZ, JL, and Z-KL. Data collection: YZ, JL, Z-KL, XY, JB, LL, TD, and GF. Data analysis: YZ, JL, and Z-KL. Manuscript preparation: YZ, JL, and Z-KL. Manuscript editing: XD and QL. All authors contributed to the article and approved the submitted version.

## Conflict of Interest

The authors declare that the research was conducted in the absence of any commercial or financial relationships that could be construed as a potential conflict of interest.

## Publisher’s Note

All claims expressed in this article are solely those of the authors and do not necessarily represent those of their affiliated organizations, or those of the publisher, the editors and the reviewers. Any product that may be evaluated in this article, or claim that may be made by its manufacturer, is not guaranteed or endorsed by the publisher.
